# Prevalence of gestational diabetes according to commonly used data sources: an observational study

**DOI:** 10.1186/s12884-019-2521-2

**Published:** 2019-10-11

**Authors:** Robyn L. Lawrence, Clare R. Wall, Frank H. Bloomfield

**Affiliations:** 1 The Liggins Institute, University of Auckland, Building 505, Level 2, 85 Park Road, Auckland, 1023 New Zealand; 2 Faculty of Medical and Health Sciences, University of Auckland, Building 505, Level 1, 85 Park Road, Auckland, 1023 New Zealand

**Keywords:** Gestational diabetes mellitus, Prevalence

## Abstract

**Background:**

It is well recognized that prevalence of gestational diabetes mellitus (GDM) varies depending on the population studied and the diagnostic criteria used. The data source used also can lead to substantial differences in the reporting of GDM prevalence but is considered less frequently. Accurate estimation of GDM prevalence is important for service planning and evaluation, policy development, and research. We aimed to determine the prevalence of GDM in a cohort of New Zealand women using a variety of data sources and to evaluate the agreement between different data sources.

**Methods:**

A retrospective analysis of prospectively collected data from the Growing Up in New Zealand Study, consisting of a cohort of 6822 pregnant women residing in a geographical area defined by three regional health boards in New Zealand. Prevalence of GDM was estimated using four commonly used data sources. Coded clinical data on diabetes status were collected from regional health boards and the Ministry of Health’s National Minimum Dataset, plasma glucose results were collected from laboratories servicing the recruitment catchment area and coded according to the New Zealand Society for the Study of Diabetes diagnostic criteria, and self-reported diabetes status collected via interview administered questionnaires. Agreement between data sources was calculated using the proportion of agreement with 95% confidence intervals for both a positive and negative diagnosis of GDM.

**Results:**

Prevalence of GDM combining data from all sources in the Growing Up in New Zealand cohort was 6.2%. Estimates varied from 3.8 to 6.9% depending on the data source. The proportion of agreement between data sources for presence of GDM was 0.70 (95% CI 0.65, 0.75). A third of women who had a diagnosis of GDM according to medical data reported having no diabetes in interview administered questionnaires.

**Conclusion:**

Prevalence of GDM varies considerably depending on the data source used. Health services need to be aware of this and to understand the limitations of local data sources to ensure service planning and evaluation, policy development and research are appropriate for the local prevalence. Improved communication of the diagnosis may assist women’s self-management of GDM.

## Background

Gestational diabetes mellitus (GDM) is frequently described as the most common metabolic disorder of pregnancy with prevalence increasing at epidemic proportions [1–3]. However, reported prevalence worldwide varies between 1 and 45% of pregnancies [4, 5]. While there are some clear reasons for this variability, others are not as obvious. Different ethnicities have different susceptibility to GDM; therefore, differences in the ethnic make-up of the population studied as well as genetic variability will result in different prevalence rates of GDM [6–9]. Similarly, the lack of consensus in which diagnostic threshold should be used to diagnose GDM results in variation in prevalence [4, 10–12]. An issue that is less frequently considered is the data source used to calculate prevalence. Population-wide cohort studies are impractical and costly; therefore, smaller cohort studies are often used to extrapolate estimates of GDM prevalence to the wider population. However, differences in the type of data used to calculate prevalence may lead to substantial differences in the reporting of GDM prevalence [13, 14]. For example, a cohort consisting of a population of women who were screened for GDM will have a smaller denominator than a cohort including all pregnant women in a given hospital in which screening of all women is not routine [14–16]. Accurate estimation of GDM prevalence is important for service planning, funding allocation, and research. Inaccurate estimates, or varied estimates within a health service due to different methodologies, may result in inequitable or inadequate care.

The prevalence of GDM in New Zealand is not definitively known and reports in the literature are from small studies in small catchment areas, with varying methodology [12, 17–21]. The Growing Up in New Zealand study is a large, ongoing, longitudinal cohort study which recruited pregnant women living within a geographical area serviced by three neighbouring regional health boards: Auckland (ADHB); Counties Manukau (CMDHB), and Waikato (WDHB) District Health Boards (DHBs), which account for almost a third of New Zealand’s population [22]. This geographical area was selected to provide a diverse birth cohort that would be broadly generalisable to New Zealand births [22]. The aim of this study was to determine the prevalence of GDM in the Growing Up in New Zealand study as a whole and according to the data source used. Prevalence of GDM in the Growing Up in New Zealand study was then compared to prevalence according to the Ministry of Health’s National Minimum Dataset.

## Methods

Data for the primary analyses in this study were derived from the Growing Up in New Zealand longitudinal study cohort, described in detail elsewhere [22]. Briefly, pregnant women estimated to birth between 25th April 2009 and 25th March 2010 and living within the geographical boundaries serviced by ADHB, CMDHB and WDHB, were invited to participate in the study. Place of residence was the only inclusion criterion and there were no exclusion criteria [22]. The need for a new birth cohort study in New Zealand was identified by the New Zealand Ministry of Social Development, which is the lead agency responsible for its commissioning and funding but which had no other role in conducting the research or in writing this manuscript. Ethical approval was obtained from the Ministry of Health Northern Y Regional Ethics Committee and written informed consent was obtained from all participating women. A total of 6822 women consented and completed the antenatal interview. Birth characteristics of the Growing Up in New Zealand cohort were comparable to national birth statistics at that time [23].

### Data sources for identification of GDM

Four data sources were used to identify cases of GDM within the cohort: coded clinical data held by the three DHBs within the study catchment area; coded clinical data held by the Ministry of Health’s National Minimum Data set; blood results including fasting plasma glucose concentration, glucose challenge test results and glucose tolerance test results obtained from laboratories servicing the recruitment catchment area; and participant self-report in the Growing Up in New Zealand antenatal and 16-month post-partum interviews. Linking to routine health records was available for women who consented to this using their unique National Hospital Identifier (NHI) (*n* = 6657). Participants’ NHIs were used to extract coding data held by DHBs and the Ministry of Health’s National Minimum Data set and blood results from laboratories servicing the recruitment catchment area. The Ministry of Health and CMDHB provided coding information according to the International Classification of Diseases (ICD) 10 codes extracted from the National Minimum Dataset and hospital wide database respectively. ADHB and WDHB provided codes as normal glucose tolerance, gestational diabetes, pre-existing type 1 diabetes, pre-existing type 2 diabetes or impaired glucose tolerance extracted from their local maternity database and diabetes clinic database respectively. Women were further coded as having GDM if they had a positive blood glucose result (at any time from 12 weeks’ gestation up until birth) according to the diagnostic criteria for GDM in use by their DHB of domicile at the time. All three DHBs in the Growing Up in New Zealand study used the New Zealand Society for the Study of Diabetes criteria [24, 25] from a 75 g oral glucose tolerance test (OGTT) to diagnose GDM: fasting plasma glucose ≥5.5 mmol/L or a 2-h plasma glucose ≥9.0 mmol/L [21, 26, 27] (Jade Tamatea, Endocrinologist, WDHB, emailed personal communication, April 27, 2017). In addition, CMDHB utilised an additional screening test, a 50 g glucose challenge test (GCT) with a single 60 min plasma glucose sample. If the result of this 60 min sample was a plasma glucose concentration ≥ 11.1 mmol/L, this was considered diagnostic of gestational diabetes without confirmation with the standard 2-h, 75 g oral glucose tolerance test [21]. Women with a plasma glucose concentrations < 11.1 mmol/L but ≥7.8 mmol/L at 60 min proceeded to a standard 75 g oral glucose tolerance test as detailed above. The pregnancy period was calculated for each woman using documented length of gestation and date of delivery. Where no length of gestation was available, 40 weeks was used as proxy (*n* = 905). Responses to the Growing Up in New Zealand antenatal and 16-month post-partum interviews were used to collate self-reported data on diabetes in pregnancy status. Participants were asked about their diabetes status in pregnancy at two time points. First, during a face-to-face computer-assisted personal interview during pregnancy (most frequently early in the third trimester) in which women were asked “Have you ever had diabetes?” with possible responses being “never”, “before this pregnancy but not during this pregnancy”, “before this pregnancy and during this pregnancy”, “only during this current pregnancy” and “don’t know”. Women were then asked again 16 months after the birth of their child(ren) in a computer-assisted telephone interview: “Thinking about the last 14 weeks of your pregnancy with [name], during this time were you diagnosed with diabetes – this would be where your doctor, midwife, or other lead maternity carer told you that you had diabetes for the first time?” Possible responses included “yes”, “no,” “don’t know”. Women who responded “only during this current pregnancy” to the first question and/or “yes” to the second question were coded as having GDM according to self-reported data. Women who had a previous pregnancy and responded “before and during this pregnancy” and “yes” to the second question were also coded as having GDM with the assumption that there was GDM in the index pregnancy and a history of GDM in a previous pregnancy. Women were coded as having GDM if they met the criteria for GDM in their DHB according to any data source. If inconsistencies were present in the type of diabetes between data sources, the most recent DHB or Ministry of Health coding data were used.

The antenatal interview also included questions about maternal socio-demographic, health, and lifestyle characteristics. Following the coding criteria used by Statistics New Zealand, self-reported ethnicity was assigned to one of six Level 1 categories: (i) European; (ii) Māori; (iii) Pacific Peoples; (iv) Asian; (v) Middle Eastern / Latin American / African (MELAA), and (vi) Other ethnicity [28]. If women identified with more than one ethnicity and did not self-prioritised a primary ethnicity, prioritisation was determined following the methodology of Statistics New Zealand in use between 1991 to 2004 [29], as a single ethnic group was required for statistical analyses. The MELAA and Other ethnicity groups were combined under the ‘Other’ category, for the statistical analyses due to small numbers in these ethnic groups. Social deprivation was measured using the New Zealand index of Deprivation (NZDep06). NZDep06 is derived from 2006 census data on nine socio-economic indicators: home ownership; household income; household crowding; access to a telephone; access to a car; single-parent family; means-tested benefits; qualifications, and employment. The index of Deprivation score is averaged for a population of a geo-coded address area with scores from 1 (least deprived 10%) to 10 (most deprived 10%) [30]. Pre-pregnancy body mass index (BMI) was calculated from self-reported pre-pregnancy height and weight.

The National Minimum Dataset is maintained by the Ministry of Health and is a national collection of public and private hospital discharge information, including coded clinical data for inpatients and day patients, and is commonly used to calculate prevalence statistics in the New Zealand health setting [31]. All hospital admissions and births occurring in New Zealand hospitals are captured by the dataset [31] and it therefore includes women enrolled in the Growing Up in New Zealand study as well as those in the general population. Data on diagnosis of GDM, DHB, age and ethnicity for all births in 2009 and 2010 were obtained from the Ministry of Health’s National Minimum Dataset and were compared with the Growing Up in New Zealand data. Calculations were made using data from the National Minimum Dataset for women from areas serviced by ADHB, CMDHB and WDHB for 2009 and 2010 combined to match the period and geographical area in which women were recruited to the Growing Up in New Zealand cohort and used to compare the prevalence of GDM in the Growing Up in New Zealand cohort obtained in this study with that obtained from the National Minimum Dataset.

### Statistical analyses

Statistical analysis was performed using SPSS version 25. Data were checked for accuracy by evaluating descriptive statistics and are reported as frequency (%) for the data available. Pearson Chi squared test and Fishers Exact test were used to analyse frequency data. The proportions of agreement between data sources were calculated according to the methods described by Grant [32] and are reported as proportion of agreement and 95% confidence interval (CI). The proportions of agreement for both presence and absence of GDM were calculated in three instances: comparing all four data sources of diabetes status (coding data from the Ministry of Health, coding data from DHBs, laboratory data and self-reported data); comparing different sources of medical data (coding data from the Ministry of Health, coding data from DHBs and laboratory data), and comparing pooled medical data to self-reported data. Statistical significance was considered at the *P* < 0.05 level. Descriptive statistics, bar charts and box plots were used to compare characteristics of women in the Growing Up in New Zealand study to the National Minimum Dataset.

## Results

**Fig. 1:**
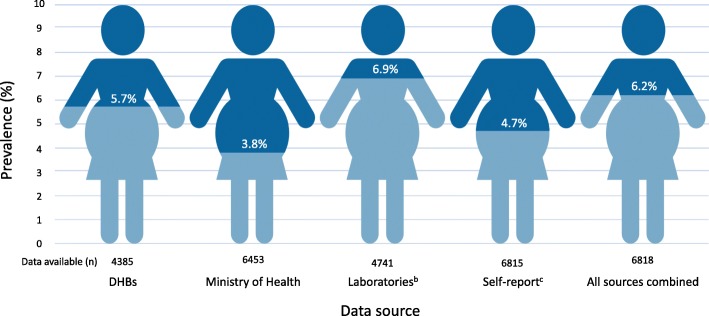
Prevalence of GDM in the Growing Up in New Zealand study according to data source. *n* number*, DHBs* District Health Boards. ^b^GDM in laboratory data defined as any positive blood glucose result after 12 weeks’ gestation in accordance with the criteria in use for each woman’s DHB of domicile during the study period. ^c^Self-reported data from antenatal and postpartum data collection points combined using responses “during this pregnancy only” and “for the first time in the last 14 weeks of pregnancy” as a proxy for GDM

The characteristics of the Growing Up in New Zealand cohort have been described previously [22, 23]. Maternal socio-demographic, health and lifestyle characteristics for women with data on diabetes status during pregnancy (*n* = 6818) are summarised in Table 1. Self-reported data were available for 6815 women and data from the Ministry of Health, DHBs, and laboratories were available for 6453, 4385, and 4741 women respectively through NHI linking. Using combined data from all data sources 67 (1.0%) women were identified as having pre-existing type 1, type 2 or impaired glucose tolerance. A diagnosis of GDM was identified in 422 (6.2%) women in the Growing Up in New Zealand cohort; however, prevalence varied depending on the data source (Fig. 1). Using medical data only i.e. data from the Ministry of Health, DHBs and laboratories, 354 (5.4%) of women were identified as having GDM (Fig. 1). Of all 422 women identified as having GDM, GDM was identified by multiple data sources for 260 (61.6%) women. Where other sources of data were either missing or did not report any presence of GDM, laboratory data exclusively identified an additional 87 (20.6%) cases, self-reported data 68 (16.1%), the Ministry of Health 4 (0.9%) and DHBs 3 (0.7%) respectively. Where data on GDM status (GDM and normal glucose tolerance) were available from multiple sources (*n* = 6483) there were conflicting data for 230 (3.6%) women. The proportion of agreement for presence of GDM was 0.70 (95% CI 0.65, 0.75) and for absence of GDM 0.98 (95%CI 0.97, 0.98) (*n* = 3840 women with data available from all four data sources). When this analysis was restricted to medical data only (*n* = 5047 with data from more than one source), 152 (3.0%) women had conflicting data from different sources. The proportion of agreement between these medical data sources for presence of GDM was 0.71 (95% CI 0.66, 0.76) and for absence of GDM 0.98 (95%CI 0.97, 0.98) (*n* = 3875 women with data available from all three medical data sources).

**Table 1:** Maternal socio-demographic, health and lifestyle characteristics for whom information on diabetes status was available

Maternal characteristic	Growing up in New Zealand (n = 6818)	National Minimum Dataset^a^ (*n* = 42,066)
	n (%)	n (%)
Age group (years)		
< 20	329 (4.8%)	2437 (5.8%)
20–24	998 (14.6%)	7715 (18.3%)
25–29	1666 (24.4%)	10,515 (25.0%)
30–34	2121 (31.1%)	11,520 (27.4%)
35–39	1419 (20.8%)	7750 (18.4%)
40 and over	285 (4.2%)	2129 (5.1%)
Self-prioritised ethnicity		
European	3608 (53.0%)	15,054 (35.8%)
Māori	950 (14.0%)	10,182 (24.2%)
Pacific	1001 (14.7%)	9355 (22.2%)
Asian	1003 (14.7%)	6498 (15.4%)
Other	241 (3.5%)	965 (2.3%)
Socioeconomic deprivation decile		
1 to 2 (least deprived)	1099 (16.1%)	
3 to 4	1235 (18.1%)	
5 to 6	1168 (17.1%)	
7 to 8	1426 (20.9%)	
9 to 10 (most deprived)	1888 (27.7%)	
Highest level of education		
No secondary school	491 (7.2%)	
Secondary school / NCEA 1–4	1627 (23.9%)	
Diploma/Trade certificate / NCEA 5–6	2082 (30.5%)	
Bachelor’s degree	1539 (22.6%)	
Higher degree	1064 (15.6%)	
DHB of domicile		
ADHB	2423 (35.5%)	13,566 (32.2%)
CMDHB	2526 (37.0%)	17,335 (41.2%)
WDHB	1869 (27.4%)	11,165 (26.5%)
Parity		
First child	2852 (41.8%)	
Pregnancy planning		
Planned	4091 (60.2%)	
Pre-pregnancy BMI (kg/m^2^)		
< 18.5	256 (4.7%)	
18.5–24.9	3261 (54.6%)	
25–29.9	1349 (22.6%)	
30 and over	1105 (18.5%)	

Data are presented as number of participants and percentages unless otherwise indicated, missing values have not been included in the column %

*n* number, *ADHB* Auckland District Health Board, *CMDHB* Counties Manukau District Health Board, *WDHB* Waikato District Health Board, *NCEA* National Certificate of Educational Achievement, *DHB* District Health Board, *BMI* Body Mass Index

^a^Data from the National Minimum Dataset for women from areas serviced by ADHB, CMDHB and WDHB for 2009 and 2010 combined to match the period and geographical area in which women were recruited to the Growing Up in New Zealand study

In cases where both self-reported and medical data were available (*n* = 6441) there was a significant discrepancy in GDM prevalence according to self-report and medical data (Table 2, *P* < 0.0005). Of these women, 176 (2.7%) gave responses to interview administered questionnaires that were inconsistent with medical data. Of the 341 women with medically-documented GDM for whom self-reported data were also available, 115 (33.7%) reported that they did not have any form of diabetes (Table 2). Of the 61 women with GDM according to self-report but no medically-documented GDM, 50 had GCT or OGTT results to suggest that they did not have GDM and none had a diagnosis of GDM coded in the DHB and Ministry of Health data. The proportion of agreement between self-reported data and medical data for a diagnosis of GDM was 0.56 (95% CI 0.51, 0.61) and for an absence of GDM 0.97 (95% CI 0.97, 0.98). Self-reported prevalence of GDM varied between the two data collection points in the interview administered questionnaires. Of 6802 women who responded to the question in the face-to-face interview administered antenatal questionnaire, 162 (2.4%) women reported having diabetes “only during this current pregnancy” and 266 (4.1%) of 6802 women replied “yes” when asked if they had diabetes diagnosed for the first time in the last 14 weeks of pregnancy in the 16-month post-partum telephone interview (*P* < 0.0005). When looking at concordance with medical data using self-reported data from each time point separately, 191 (54.3%) of the 352 women with medically-documented GDM reported “never” having diabetes in the antenatal questionnaire and 142 (42.4%) of 335 women with medically documented GDM reported “no” when asked if they had diabetes diagnosed for the first time in the last 14 weeks of pregnancy in the 16-month post-partum telephone interview.

**Table 2:** GDM status according to self-reported^a^ and medical^b^ data in Growing Up in New Zealand

Self-reported GDM status^a^	GDM status according to medical data^b^		*P*-value
	Normal glucose tolerance	GDM	
No diabetes	6039 (93.7%)	115 (1.7%)	< 0.0005
GDM	61 (0.9%)	226 (3.5%)	

Data are presented as number of participants and percentages unless otherwise indicated, missing values have not been included in the column %. Women who were identified as having other forms of diabetes either by self-report or medical data (*n* = 113) were excluded from this table. Distributions are compared by Pearson chi-square test

*GDM* gestational diabetes mellitus, *n* number

^a^Self-reported data from antenatal and postpartum data collection points combined using responses “during this pregnancy only” and “for the first time in the last 14 weeks of pregnancy” as a proxy for GDM

^b^Medical data combines data from the District Health Boards, Ministry of Health and laboratories

The National Minimum Dataset has 42,066 live births recorded for ADHB, CMHDB, and WDHB for 2009 and 2010. Of these, 1552 (3.7%) mothers were coded as having GDM during pregnancy. Maternal characteristics of women in the National Minimum Dataset from ADHB, CMDHB and WDHB for 2009 and 2010 are shown in Table 1 and are comparable to that of women in the Growing Up in New Zealand study.

## Discussion

### Main findings

The prevalence of GDM in the Growing up in New Zealand study varied significantly between data sources. Using data from all sources, GDM prevalence was 6.2%. When this analysis was restricted to medical data only, GDM prevalence was 5.4%. The prevalence of GDM found in the Growing Up in New Zealand study cohort was 68% greater than the prevalence from the National Minimum Dataset for the same geographical area during the same time period.

Where data from the Growing Up in New Zealand cohort were available from multiple sources, data were conflicting for 3.6% of women and levels of agreement for a diagnosis of GDM were poor. We found discrepancies in self-reported data when compared to medical data in which a third of women with a diagnosis of GDM according to medical data reported having no diagnosis of diabetes in self-reported data.

### Interpretation

Diagnosis of GDM according to medical records is frequently considered to be a gold-standard data source estimating the prevalence of GDM in a population [33–35]; however, review of medical records is labour-intensive, expensive and access to records restrictive. Population health datasets are frequently used to determine disease prevalence and are derived from coding of medical diagnoses present in clinical records [31], but their accuracy has been questioned [21, 35]. Self-reported data have been suggested to be an accurate alternative data source for estimating the prevalence of GDM [33, 36, 37]. However, the substantial differences in GDM prevalence seen according to different data sources in the Growing Up in New Zealand study and between the Growing Up in New Zealand cohort and the National Minimum Dataset highlight significant deficiencies in using just one data source to determine GDM prevalence. Where data were available from multiple sources, data were conflicting for 3.6% women and levels of agreement between data sources for presence of GDM were poor.

Other studies evaluating the prevalence of GDM in routinely collected population health datasets have shown similar findings [34, 35, 38, 39]. Zheng, Morris and Moses [38] determined the prevalence of GDM in a private hospital according to the hospital’s records and laboratory results and compared this to the New South Wales Perinatal Data collection. Much like the findings in our study, there were discrepancies in GDM prevalence according to different data sources and both hospital records and the Perinatal Data collection underestimated the prevalence of GDM. For women who were missing a diagnosis of GDM in the Perinatal Data collection, about half had a diagnosis of GDM documented in the medical records and half were not documented in the women’s medical notes [38]. Bell et al. [34] compared information on maternal diabetes status extracted from medical records of a random sample of 1200 women giving birth in New South Wales, Australia and compared this to two New South Wales Department of Health routinely collected datasets. Both datasets underestimated the prevalence of GDM when compared to medical records and given the findings of Zheng, Morris and Moses [38], where half the cases of GDM were not documented in the medical notes, the discrepancy between the prevalence of GDM recorded in the datasets and the true prevalence of GDM could in fact be even greater.

Other studies have suggested self-reported data provide an accurate estimate of GDM prevalence [33, 36, 37]. Gresham et al. [37] investigated the agreement between self-reported perinatal outcomes, collected through repeated surveys, and medical records in the Australian Longitudinal Study on Women’s Health. When women were asked specifically about each of their pregnancies, there was an agreement of 97.8%, Kappa 0.66 (*P* < 0.001) between self-reports and medical records for GDM [37]. Similarly, in the New York State Pregnancy Risk Assessment Monitoring System (PRAMS) study, Hosler, Nayak and Radigan [33] examined agreement between participating women’s self-report and maternal GDM documented on their children’s birth certificates and found percent agreement to be 93.8% with a Kappa statistic of 0.53. Despite these seemingly high levels of agreement, the Kappa statistic used in these studies is testing the correlation between the two reports of GDM, but does not test their level of agreement [32]. Using the data provided by Gresham et al. [37] the proportions of agreement between self-reported data and medical records can be calculated to be 0.51 (95% CI 0.47, 0.55) for the presence of GDM and 0.98 (95% CI 0.97, 0.98) for the absence of GDM, very similar to our findings. These data also show that 2.2% of women misreported their GDM status according to medical records in the study by Gresham et al. [37], comparable to the 2.7% found in our study, and 6.2% of women misreported their GDM status in the study by Hosler, Nayak and Radigan [33]. These results question the validity of using self-report as the only data source for estimating GDM prevalence. More importantly, any number of women who misinterpret their diagnosis is likely to have unfavourable consequences. Appropriate treatment of GDM, even in mild cases, has been shown to reduce the risk of adverse pregnancy outcomes [40]. Our finding that a third of women with a diagnosis of GDM according to medical data did not report having any form of diabetes when asked in interview administered questionnaires raises the question as to whether these women received or adhered to treatment for GDM and warrants further investigation. The greater proportion of women reporting to have GDM and lower incidence of misreporting their diagnosis when compared to medical data at the post-partum time point compared to the antenatal time point could be due to women being diagnosed with GDM after the antenatal questionnaire but could also be due to the difference in interview technique used.

Researchers, healthcare organisations, policy makers and funders rely on prevalence statistics for service planning, policy development and funding allocation. The findings in our study and others’ [34, 38, 39] indicate that commonly used prevalence statistics are likely underestimating the true prevalence of GDM. By using multiple data sources to determine GDM prevalence, we were less likely to miss any diagnoses of GDM and therefore give a more accurate estimate of GDM prevalence.

### Strengths and limitations

To our knowledge this is the first study evaluating the proportions of agreement between different data sources for the presence and absence of GDM in a population. Although effort was made to have a consistent approach to data collection, not all DHBs provided the same type of information when diabetes coding status was requested using NHI linking. CMDHB provided data on diabetes coding based on ICD-10 codes from their hospital database, while ADHB provided data extracted from their maternity database, and WDHB matched NHIs to their diabetes clinic database and therefore only provided information on women who were registered with the diabetes clinic resulting in a significant number of missing data from ADHB and WDHB. Furthermore, while all three DHBs used a 75 g OGTT with the same fasting and 2-h plasma glucose thresholds for diagnosis as their formal diagnostic test, CMDHB additionally used a 50 g screening test for which a plasma glucose concentration at 60 min of ≥11.1 mmol/L was considered diagnostic of GDM [21]; thus, the diagnosis of GDM was not made consistently across the cohort. The nature of the different data sources give different denominators when calculating prevalence. For example, the laboratory data includes only those women who were screened for GDM, whilst the Ministry of Health National Minimum Dataset includes all women who delivered at a New Zealand Hospital. Furthermore, although the self-reported data included data collected from more than one time point, the wording used in the interview administered questionnaires did not specifically ask about GDM per se and could be open to interpretation and misclassification in coding. The participants’ understanding of these questions could also be influenced by factors such as level of education, the care they received during pregnancy and pregnancy outcome, and may have affected their responses. While these differences may limit the robustness of the data, a major strength of our study is that by pooling results from multiple data sources, we were able to overcome the deficiencies of the different data types to give a more accurate estimate of GDM prevalence. An additional strength is that the prevalence of GDM calculated from NHI linked data from the Ministry of Health of 3.8% was almost identical to the 3.7% prevalence found in the National Minimum Dataset for the same geographical area. This suggests that the cohort of women in the Growing Up in New Zealand study were broadly representative, at least with respect to risk factors for GDM, to all women giving birth in the catchment area at the time. We acknowledge that the data used to determine prevalence of GDM in this cohort were collected 10 years ago and may not reflect current GDM prevalence. However, to date this is the largest study to estimate GDM prevalence in New Zealand and provides a reference for future research and raises important points to consider when utilising or collecting prevalence statistics.

## Conclusions

Our results suggest that the true prevalence of GDM is likely to be different to that commonly reported in the literature, particularly when only one data source is used to determine prevalence. Given that prevalence of GDM varies considerably depending on the data source, this needs to be taken into consideration when evaluating prevalence of GDM and researchers should consider using more than one data source to determine the prevalence of GDM in a population. Inaccuracies in prevalence data are likely to have significant implications for service planning and evaluation, policy development and research. A large proportion of women in New Zealand appear to be unaware of their diagnosis of GDM and thus self-report should not be used to estimate prevalence. Lack of awareness of the diagnosis may impact negatively on a woman’s ability to manage GDM and, therefore, potentially on pregnancy outcomes for her and her baby. This discrepancy is concerning and warrants further investigation into communication of the diagnosis to affected women.

## Data Availability

The data that support the findings of this study are available from Growing Up in New Zealand and the New Zealand Ministry of Health but restrictions apply to the availability of these data, which were used under license for the current study, and so are not publicly available. Data are, however, available from the authors upon reasonable request and with permission of Growing Up in New Zealand and the New Zealand Ministry of Health.

## Abbreviations

ADHB: Auckland District Health Board
CI: Confidence interval
CMDHB: Counties Manukau District Health Board
DHB: District Health Board
GCT: glucose challenge test
GDM: Gestational diabetes mellitus
ICD-10: International Classification of Diseases 10
NHI: National Hospital Identifier
NZDep06: New Zealand index of Deprivation
OGTT: Oral glucose tolerance test
WDHB: Waikato District Health Board
